# The perceived levels of stress, anxiety and depression among family caregivers of patients undergoing haemodialysis and their association with quality of life

**DOI:** 10.1192/bjo.2025.44

**Published:** 2025-05-13

**Authors:** Aisha Al Maqbali, Omar Al Omari, Loai Abu Sharour, Zakariya Al-Naamani, Mahmood Al Khatri, Hala Mohamed Sanad, Iman Al Hashmi, Abdullah Alkhawaldeh, Mohammad Al Qadire, Domam Al Omari

**Affiliations:** Faculty of Nephrology programme, Higher Institute of Health Specialties, Muscat, Oman; College of Nursing, Sultan Qaboos University, Muscat, Oman; Higher Colleges of Technology, Ras Al-Khaimah, UAE; Medical City for Military and Security Services School, Ministry of Defense, Muscat, Oman; Ministry of Health, Muscat, Oman; Department of Nursing, University of Bahrain, Zallaq, Kingdom of Bahrain; Princess Salma Faculty of Nursing, Al Al-Bayt University, Mafraq, Jordan

**Keywords:** Anxiety, stress, caregivers, haemodialysis, renal failure

## Abstract

**Background:**

Acknowledging the impact of chronic kidney disease on caregivers’ quality of life (QoL) and psychological well-being has become a global priority, highlighting the need for supportive interventions specifically aimed at caregivers.

**Aims:**

This study aimed to assess the prevalence of stress, anxiety and depression among family caregivers of Omani patients undergoing haemodialysis and to explore its association with QoL.

**Method:**

The study employed a cross-sectional design. A sample of 326 participants completed the study’s surveys, including the Depression Anxiety Stress Scale, WHOQOL-BREF scale and a demographic scale.

**Results:**

The survey indicated that 68.4% of the participant caregivers experienced varying degrees of depression. In addition, 48.4% of caregivers reported experiencing stress levels ranging from mild to extremely severe. For anxiety, 65.6% (*n* = 214) of caregivers noted varying levels, from mild to extremely severe anxiety. Significant negative associations were found among caregiver age, number of chronic illnesses, number of medications, daily hours spent on caregiving, physical health, stress, anxiety and depression, on the one hand, and the physical domain of QoL, on the other hand. Regarding the psychological domain of QoL, significant negative associations were observed with daily caregiving hours, physical health, stress, anxiety and depression.

**Conclusion:**

This study highlights the significant psychological burden faced by caregivers of patients undergoing haemodialysis. Systematic screening and practical interventions, such as support groups and mental health programmes, are essential to improve caregiver well-being. Future research should explore the effectiveness of these interventions and the long-term impact of caregiving.

Chronic kidney disease (CKD) is recognised as a significant health concern because of its chronic nature and the extensive treatment it necessitates.^1^ The global incidence of CKD has consistently increased, from an estimated 843.6 million individuals in 2013 to 860.8 million in 2017.^2^ CKD is a progressive disease that can deteriorate into kidney failure, necessitating a kidney transplant or haemodialysis for survival. In Oman, the Ministry of Health reported that 2588 individuals were receiving haemodialysis in 2023, indicating a steady increase in the number of patients undergoing kidney therapies over the last 30 years.^3^ Patients with CKD often depend on advanced medical equipment, intricate procedures and a dedicated team of healthcare professionals for their lifelong care.^4^ Treatment typically involves prolonged sessions of haemodialysis, usually lasting about 4 h each and occurring two to four times a week, depending on the patient’s specific needs. These sessions can be both monotonous and demanding for patients and their primary caregivers alike.^4,5^

## Literature review

Several studies have explored the relationship between caregiver burden and psychological issues, focusing on stress, depression and anxiety.^1,6–8^ A notable study in Saudi Arabia assessed the prevalence of depression among caregivers of patients undergoing haemodialysis, finding that 53% exhibited symptoms of depression.^1^ Among these, 31% experienced mild depression, 14% had moderate depression and 8% exhibited severe depression, with none reaching very severe levels. Similarly, a Jordanian study evaluated the impact of depression and anxiety on the quality of life (QoL) of family caregivers and patients with end-stage kidney disease (ESKD), finding comparable anxiety levels but higher depression in patients than caregivers.^6^ Research shows that caregivers of patients with ESKD often face significant psychological challenges.^7,9^ One study investigating psychological distress among patients with ESKD and their caregivers reported that 66.7% of the 34 participating caregivers experienced stress, highlighting concerns such as anxiety, depression and fatigue.^7^ These findings emphasise the need for further investigation into the psychological and QoL challenges faced by caregivers of patients undergoing haemodialysis, particularly in regions like Oman where such issues remain underexplored. A deeper understanding of the negative consequences experienced by these caregivers can guide the development of effective interventions and support systems aimed at reducing their burden and improving their overall well-being and QoL.^10,11^

A study in China assessed the QoL of 407 primary family caregivers of chronically ill elderly individuals. Results indicated that family caregivers faced a decline in many aspects of their QoL. Factors influencing this decline included the duration of caregiving and the financial burdens of healthcare.^12^ In Iran, the length of caregiving per day was another significant negative predictor of poor physical QoL.^13^ In addition, a Turkish study compared the QoL among caregivers of patients undergoing different kidney treatments, finding that caregivers of patients undergoing haemodialysis scored lowest on the WHOQOL-BREF scale.^14^

The impact of CKD on caregivers’ QoL and psychological well-being has become a global concern, highlighting the need for supportive interventions specifically aimed at caregivers. While much research has focused on Western populations, the experiences of caregivers in the Arab world, and specifically in Oman, have received less attention. Currently, there is a noticeable gap in research examining the relationship among stress, anxiety and depression, on the one hand, and QoL, on the other, among caregivers of patients undergoing haemodialysis in Oman.

## Method

### Design and sampling

The study employed a cross-sectional design with a convenience sampling approach. The eligibility criteria included adult caregivers aged 18 years or older who were first- or second-degree relatives of patients and had provided care for the patient for at least 3 months. The 3-month minimum care duration criterion was established to account for the initial clinical instability of patients new to haemodialysis,^15^ caused by the invasive procedure for vascular access creation, frequent admissions to hospital and an increased risk of mortality. Caregivers with mental illness were excluded from the current study. This was assessed directly during the pre-enrolment process by asking the potential participants: ‘Do you have a diagnosed mental illness, or have you visited a psychologist or general practitioner for issues related to mental illness?’ Those who responded ‘yes’ were excluded. However, as this screening took place at haemodialysis centres before formal enrolment, we could not ascertain the number of individuals excluded.

The researchers used G*Power (version 3.1.9.7 for Windows; Heinrich-Heine-Universität Düsseldorf, Düsseldorf, Germany; https://www.psychologie.hhu.de/arbeitsgruppen/allgemeine-psychologie-und-arbeitspsychologie/gpower) to calculate the sample size with the following parameters: alpha set at 0.05, power at 80% and a moderate effect size of 0.25. Based on the calculation, the final sample size was 320 participants. However, in the current study 326 participants were recruited from various haemodialysis centres in the North Batinah and Muscat Governorate regions. These centres were selected due to their large out-patient populations from diverse geographical areas.

### Ethical approval

Ethical approval for this study was obtained from the Nursing College at Sultan Qaboos University (CON/MSN/2021/5) and the Research and Ethics Committee at the Ministry of Health (MoH/CSR/21/25358). Administrative permissions were also granted by the hospital administration and nursing heads. The study adhered to guidelines for ethics, privacy and confidentiality. Data was securely stored, and completed surveys were kept locked. This study adhered to the Declaration of Helsinki.

### Data collection

After obtaining ethical and administrative approval, researchers approached the haemodialysis centres and potential participants were identified through patient registers. Eligible caregivers were invited to participate, and detailed information about the study was provided to the potential participants. The inclusion and exclusion criteria were clearly outlined in the information sheet and consent form and explained by research assistants during the recruitment process.

To determine whether caregivers had a history of mental illness, they were asked a straightforward question during the pre-enrolment process: ‘Do you have a diagnosed mental illness or have you visited a psychologist or general practitioner for issues related to mental illness?’ Those who responded ‘yes’ were not eligible to participate in the study. This question was clearly stated in the consent form and information sheet provided to participants, ensuring that the criteria were transparent and easy to understand.

However, since this screening was conducted at haemodialysis centres before formal enrolment, we were unable to track the exact number of individuals excluded because of mental illness or other reasons. Similarly, we could not document the total number of people approached or those who chose not to participate. We recognise this as a limitation in our ability to provide detailed figures regarding recruitment and exclusions.

Despite these constraints, our recruitment strategy and inclusion criteria were carefully designed to ensure that the final sample was representative of caregivers actively supporting patients undergoing haemodialysis. These efforts aimed to minimise any potential selection bias and provide findings that genuinely reflect the experiences of this caregiving population.

Lastly, caregivers who met the inclusion criteria and agreed to participate signed a consent form and completed the survey.

### Measurement

#### Psychological distress

The Depression Anxiety Stress Scale (DASS) is designed to assess emotional distress in adults across three subcategories: depression, anxiety and stress. Each subcategory comprises seven items, and participants rate their responses using a Likert scale ranging from 0 to 4. The scores for each subcategory are summed up and multiplied by two, with a maximum possible score of 42. The scores for each subcategory are summed, and participants are classified into severity ranges: normal, mild, moderate, severe and extremely severe. The cut-off scores for each category are as follows.^16^

- **Depression**: normal (0–9), mild (10–13), moderate (14–20), severe (21–27), extremely severe (28+).
- **Anxiety**: normal (0–7), nild (8–9), moderate (10–14), severe (15–19), extremely severe (20+).
- **Stress**: normal (0–14), mild (15–18), moderate (19–25), severe (26–33), extremely severe (34+).

These cut-off scores were used to classify participants’ symptom severity in the current study. Higher scores indicate greater emotional distress, while lower scores reflect less severe symptoms. The DASS has shown strong internal consistency and validity, as evidenced by high coefficient alpha values for the stress, anxiety and depression scales.^17^ It has also been translated into Arabic, showing good reliability indicated by Cronbach’s alpha coefficients of 0.83^18^ and 0.88^19^ in different studies. The DASS is available for public use.

It is important to note that the DASS is not intended to diagnose clinical mental health disorders but rather to assess the severity of emotional symptoms. This sensitivity makes it particularly useful for identifying distress in populations experiencing chronic stress, such as caregivers of patients undergoing haemodialysis. The caregiving role often involves long hours, emotional strain and limited external support, all of which can elevate distress levels even in the absence of diagnosed mental illness.

### Quality of life

QoL was measured using the WHOQOL-BREF scale, a 26-item self-report questionnaire that serves as a condensed version of the WHOQOL-100 scales.^20^ It assesses six dimensions of QoL: physical health, psychological health, level of independence, social interactions, environment and spirituality.^21^ The scale has demonstrated good psychometric properties, with high coefficient alpha values indicating good reliability.^20,21^ In the context of Kuwait, the Arabic version of the WHOQOL-BREF questionnaire exhibited good internal consistency, with Cronbach’s alpha values exceeding 0.7 for all subscales.^22^ The WHOQOL-BREF scale is available for public use.

### Data analysis

The Statistical Package for Social Sciences (SPSS) version 23 for Windows (IBM Corp., Armonk, NY, USA) was used to analyse the data. Both descriptive and inferential statistics were employed to address the research questions. Parametric tests were conducted, as the data were normally distributed. Pearson’s correlation coefficients were employed to examine the relationships between various continuous variables. Mean comparisons were conducted using two-sample *t*-tests or one-way analysis of variance (ANOVA). The significance level for statistical tests was set at α = 0.05.

## Results

The mean age of caregivers was 39.8 years (s.d. = 10.85, range 18–72). Two-thirds were first-degree relatives (*n* = 210, 64.4%) and did not receive any financial support (*n* = 281, 86.2%). Most caregivers reported not having a nurse or assistant at home to care for the patient (*n* = 320, 98.2% and *n* = 289, 88.7%, respectively). The mean duration of caregiving was 6.38 years (s.d. = 5.15), and the mean daily hours of caregiving were 10.63 h (s.d. = 8.62). Further details on the caregivers’ characteristics are provided in Table 1.

**Table 1:** Demographic characteristics of caregivers (*n* = 326)

Characteristics	*n*	%		*n*	%
Gender			Taking care alone		
Male	166	50.9	Yes	179	54.9
Female	160	49.1	No	147	45.1
Level of education			Employment		
High school or less	195	59.8	Government employee	88	27
Diploma	81	24.8	Unemployed/retired	203	62.3
Bachelor	50	15.3	Private employee	35	10.7
Relationship to patient			Physical health		
Husband/wife	62	19	Excellent	77	23.6
Son/daughter	210	64.4	Very good	92	28.2
Son/daughter-in-law	13	4	Good	90	27.6
Siblings	29	8.9	Neither good nor bad	58	17.8
Parents	12	3.7	Bad	9	2.8
Assistant for patient			Assistance at home		
Yes	37	11.3	Yes	103	31.6
No	289	88.7	No	223	68.4
Nurse at home			Chronic illness – HTN		
Yes	6	1.8	Yes	60	18.4
No	320	98.2	No	266	81.6
Chronic illness – heart disease			Chronic illness – diabetes mellitus		
Yes	6	1.8	Yes	45	13.8
No	320	98.2	No	281	86.2
Financial support			Living in the same house		
Yes	45	13.8	Yes	259	79.4
No	281	86.2	No	67	20.6
Monthly income			The activity of daily living		
Less than 500 OMR	209	64.1	Alone	253	77.6
500–1000 OMR	86	26.4	Need assistance	73	22.4
More than 1000 OMR	31	9.5	Marital status		
	Mean	s.d.	Single	71	21.8
Age	39.87	10.85	Married	231	70.9
Number of chronic illnesses	0.466	0.87	Divorced	16	4.9
Medication number	0.515	1.03	Widowed	8	2.5
Years taking care	6.38	5.15			
Daily hours taking care	10.63	8.62			
Distance to dialysis centre (in km)	28.58	24.51			

HTN, hypertension; OMR, Omani rials.

### The level of QoL and psychological distress among caregivers

Among the caregiver participants, 68.4% (*n* = 223) experienced varying degrees of depression, ranging from mild to extremely severe. Specifically, 12.6% (*n* = 41) had mild depression, while 30.7% (*n* = 100) had extremely severe depression. In addition, 48.4% (*n* = 158) of caregivers reported experiencing stress levels ranging from mild to extremely severe. Among these, 15% (*n* = 49) experienced moderate stress, while 13.2% (*n* = 43) experienced severe stress. For anxiety, 65.6% (*n* = 214) of caregivers indicated varying levels of anxiety, from mild to extremely severe anxiety, with an alarming 31.9% (*n* = 104) experiencing extremely severe anxiety. Furthermore, the data indicated that the average score for the social relationship domain of QoL was 64.3 (s.d. = 25.6), while the psychological domain averaged 61.8 (s.d. = 21.1). The lowest average score was observed in the physical domain (mean 52.2, s.d. = 23.8), as shown in Table 2.

**Table 2:** Levels of quality of life (QoL), depression, anxiety and stress

Domain	Level	Mean	s.d.	Range (min–max)
QoL – Physical		52.2	23.8	0–100
QoL – Psychological		61.8	21.1	0–100
QoL – Social		64.3	25.6	0–100
QoL – Environmental		58.1	21.3	0–100
		*n*	%	
Stress				
	Normal	168	51.5	
	Mild	30	9.2	
	Moderate	49	15	
	Severe	43	13.2	
	Extremely severe	36	11	
Depression				
	Normal	103	31.6	
	Mild	41	12.6	
	Moderate	50	15.3	
	Severe	32	9.8	
	Extremely severe	100	30.7	
Anxiety				
	Normal	112	34.4	
	Mild	21	6.4	
	Moderate	57	17.5	
	Severe	32	9.8	
	Extremely severe	104	31.9	

QoL, quality of life.

### Bivariate analysis

#### Physical QoL

A significant difference was observed in caregivers based on whether they received assistance with daily living activities. Caregivers who received assistance reported a significantly lower physical QoL (mean 41.38, s.d. = 18.64) compared to those who managed activities independently (mean 55.35, s.d. = 24.25, *P* = 0.003). In addition, caregivers with servants for the patient reported a significantly higher physical QoL (mean 54.53, s.d. = 27.53) compared to those without such assistance (mean 51.92, s.d. = 23.33, *P* = 0.04; see Table 3). Significant negative associations were also found between the physical domain of QoL and various factors, including caregiver age (*r* = −0.122, *P* = 0.02), number of chronic illnesses (*r* = −0.14, *P* = 0.01), number of medications (*r* = −0.14, *P* = 0.01), daily hours spent on caregiving (*r* = −0.14, *P* = 0.01), physical health (*r* = −0.32, 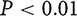 ), stress (*r* = −0.54, 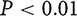 ), anxiety (*r* = −0.52, 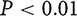 ) and depression (*r* = −0.50, 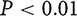 ; see Table 4).

**Table 3:** Differences in mean quality of life (QoL) among caregivers’ characteristics

	QoL – Physical		QoL – Psychological		QoL – Social		QoL – Environmental	
Characteristic	Mean (s.d.)	*P*	Mean (s.d.)	*P*	Mean (s.d.)	*P*	Mean (s.d.)	*P*
Gender		0.31		0.84		0.74		0.51
Male	51.16 (23.06)		62.50 (20.63)		62.95 (25.05)		58.71 (20.98)	
Female	53.32 (24.60)		61.11 (21.67)		65.83 (26.23)		57.48 (21.80)	
Living with patient		0.19		**0.02**		**0.04**		**0.04**
Yes	51.75 (24.27)		61.63 (21.88)		62.93 (26.15)		57.61(22.03)	
No	54.05 (22.03)		62.56 (18.02)		69.90 (22.88)		60.02 (18.60)	
Taking care alone		0.97		0.44		0.27		0.11
Yes	50.25 (23.91)		60.47 (21.82)		62.01 (25.92)		55.83 (21.89)	
No	54.61 (23.55)		63.46 (20.19)		67.23 (25.08)		60.88 (20.44)	
Financial support		0.80		0.49		0.81		0.16
Yes	55.23 (25.39)		64.25 (23.77)		67.77 (27.27)		60.48 (25.13)	
No	51.74 (23.56)		61.43 (20.69)		63.81 (25.38)		57.72 (20.73)	
Do you have diabetes mellitus?		0.35		**0.05**		0.63		**0.01**
Yes	44.37 (21.61)		63.20 (17.03)		61.70 (25.33)		58.11 (16.10)	
No	53.54 (23.95)		61.58 (21.75)		64.81 (25.71)		58.10 (22.15)	
Do you have HTN?		0.51		**0.02**		0.53		0.06
Yes	50.00 (24.64)		65.20 (16.85)		66.11 (23.10)		59.58 (18.42)	
No	52.72 (23.64)		61.05 (21.93)		63.97 (26.20)		57.77 (21.99)	
Do you have haemodialysis?		0.09		0.39		0.71		0.48
Yes	26.19 (14.04)		51.38 (19.12)		54.16 (22.82)		56.25 (17.89)	
No	52.71 (23.70)		62.01 (21.14)		64.55 (25.68)		58.14 (21.45)	
Nurse at home		0.07		0.83		0.99		0.71
Yes	51.78 (34.08)		70.13 (22.26)		80.55 (26.17)		68.22 (20.09)	
No	52.23 (23.65)		61.66 (21.11)		64.06 (25.57)		57.91 (21.37)	
Servant for patient		**0.04**		0.75		0.09		0.36
Yes	54.53 (27.53)		65.42 (21.26)		74.77 (22.94)		69.00 (19.36)	
No	51.92 (23.33)		61.36 (21.10)		63.03 (25.69)		56.71 (21.24)	
Servant at home		0.47		0.12		0.51		0.28
Yes	50.83 (24.37)		63.51 (19.70)		66.18 (25.07)		62.62 (19.85)	
No	52.86 (23.58)		61.04 (21.75)		63.52 (25.91)		56.02 (21.76)	
Activity of daily living		**0.003**		0.16		0.26		0.17
Alone	55.35 (24.25)		62.69 (21.48)		66.00 (25.10)		59.98 (21.83)	
Assistance	41.38 (18.64)		58.79 (19.67)		58.67 (26.83)		51.62 (18.38)	
Marital status		0.51		0.50		0.60		0.42
Single	54.17 (23.59)		58.68 (21.85)		63.14 (28.05)		54.53 (22.60)	
Married	51.51 (23.81)		62.78 (20.62)		65.18 (24.80)		59.30 (20.99)	
Divorce	57.58 (26.17)		63.54 (24.65)		56.77 (26.03)		57.61 (22.87)	
Widowed	44.64 (21.84)		58.33 (22.71)		66.66 (28.52)		56.25 (17.67)	
Education level		**0.04**		**0.02**		0.06		**0.002**
High school or less	49.61 (23.45)		59.25 (21.21)		61.62 (25.87)		54.75 (21.39)	
Diploma	54.85 (24.82)		65.02 (20.87)		68.51 (26.31)		62.07 (21.08)	
Bachelor	58.14 (22.47)		66.66 (20.02)		68.33 (22.46)		64.75 (19.36)	
Employment		**0.03**		0.08		0.43		**0.02**
Governmental employee	56.65 (24.13)		65.48 (21.59)		66.95 (25.18)		62.96 (22.58)	
Unemployed/retired	49.36 (23.83)		59.68 (20.77)		62.87 (26.29)		55.49 (20.70)	
Private sector employee	55.54 (21.83)		63.65 (21.02)		65.60 (23.80)		59.64 (20.38)	
Monthly income		**0.01**		**0.01**		**0.03**		**<0.01**
<500 OMR	49.36 (23.04)		59.40 (21.21)		61.72 (26.40)		54.11 (20.86)	
500–1000 OMR	57.64 (24.47)		67.29 (20.64)		69.96 (22.48)		65.47 (20.31)	
>1000 OMR	56.45 (24.61)		62.90 (19.46)		66.66 (26.70)		64.61 (21.15)	
Relationship with patient		0.35		0.32		0.37		0.84
Husband/wife	52.82 (21.73)		64.04 (22.34)		67.47 (24.55)		58.26 (21.35)	
Son/daughter	50.62 (24.37)		60.73 (20.38)		63.21 (25.23)		57.79 (21.35)	
Son/daughter-in-law	54.12 (23)		60.89 (22.72)		60.89 (32.52)		55.76 (26.87)	
Siblings	57.90 (22.34)		60.56 (21.91)		62.50 (27.63)		58.14 (21.08)	
Parents	60.98 (27.37)		72.43 (23.35)		75.64 (25.33)		64.66 (18.14)	

HTN, hypertension; OMR, Omani rials.

Bold entries mean the results are significantly less than 0.05.

**Table 4:** Pearson’s *r* correlation coefficient between quality of life (QoL) and other continuous variables related to caregivers (*n* = 326)

Caregiver’s characteristics	QoL – Physical		QoL – Psychological		QoL – Social		QoL – Environmental	
	(Pearson’s *r*)	*P*	(Pearson’s *r*)	*P*	(Pearson’s *r*)	*P*	(Pearson’s *r*)	*P*
Age	−0.122	**0.02**	−0.015	0.79	0.001	0.99	−0.005	0.92
Number of chronic illnesses	−0.14	**0.01**	−0.020	0.72	−0.032	0.56	−0.022	0.69
Number of medications	−0.11	**0.03**	0.002	0.97	0.032	0.56	0.026	0.64
Years taking care	−0.40	0.47	−0.053	0.33	−0.011	0.85	0.003	0.95
Daily hours taking care	−0.133	**0.01**	−0.13	**0.01**	−0.089	0.11	−0.055	0.32
Distance to the dialysis centre	−0.17	0.18	−0.04	0.44	−0.125	**0.02**	−0.128	**0.02**
Physical health	−0.32	**<0.01**	−0.33	**<0.01**	−0.205	**<0.01**	−0.298	**<0.01**
Stress	−0.54	**<0.01**	−0.44	**<0.01**	−0.362	**<0.01**	−0.325	**<0.01**
Anxiety	−0.52	**<0.01**	−0.46	**<0.01**	−0.364	**<0.01**	−0.344	**<0.01**
Depression	−0.50	**<0.01**	−0.48	**<0.01**	−0.394	**<0.01**	−0.369	**<0.01**
QoL-Q1	0.50	**<0.01**	0.68	**<0.01**	0.546	**<0.01**	0.659	**<0.01**
QoL-Q2	0.49	**<0.01**	0.58	**<0.01**	0.429	**<0.01**	0.506	**<0.01**

QoL-Q1, How would you rate your quality of life? QoL-Q2, How satisfied are you with your health?

Bold entries mean the results are significantly less than 0.05.

#### Psychological QoL

Several caregiver characteristics were associated with significant differences in psychological QoL. Caregivers who did not live with the patient reported a higher psychological QoL (mean 62.56, s.d. = 18.02) than those who did (mean 61.63, s.d. = 21.88, *P* = 0.02). The presence of diabetes mellitus was also significant, with caregivers without diabetes mellitus reporting a lower psychological QoL (mean 61.58, s.d. = 21.75) compared to those with diabetes mellitus (mean 63.20, s.d. = 17.03, *P* = 0.05). Other caregiver characteristics associated with significant differences in psychological QoL included education level (*P* = 0.02) and monthly income (*P* = 0.01), indicating the significant impact of these variables on psychological well-being (see Table 3). Furthermore, the number of daily hours spent on caregiving exhibited a weak negative correlation with psychological QoL (*r* = −0.13, *P* = 0.01), indicating that more caregiving hours were associated with lower psychological QoL. Physical health showed a moderate negative correlation with psychological QoL (*r* = −0.33, 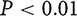 ). Stress, anxiety and depression all exhibited strong negative correlations with psychological QoL (*r* = −0.44, 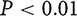 ; *r* = −0.46, 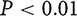 ; and *r* = −0.48, 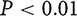 , respectively; see Table 4).

#### Social QoL

Living with the patient significantly affected caregivers’ social QoL. Caregivers who did not live with the patient reported higher scores (mean 69.90, s.d. = 22.88) compared to those who did (mean 62.93, s.d. = 26.15, *P* = 0.04). In addition, socialisation played a significant role, as caregivers with social support reported higher social QoL (mean 69.90, s.d. = 22.88, *P* = 0.006). Other caregiver characteristics associated with significant differences in social QoL included caregiver monthly income (*P* = 0.03), having a servant (*P* = 0.09) and having chronic conditions (*P* = 0.002; see Table 3). Social QoL was significantly negatively correlated with the distance to the dialysis centre (*r* = −0.125, *P* = 0.02), suggesting that caregivers living farther from the centre had lower social QoL. Physical health was also negatively correlated with social QoL (*r* = −0.205, 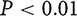 ), as were stress (*r* = −0.362, 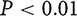 ), anxiety (*r* = −0.364, 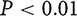 ) and depression (*r* = −0.394, 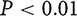 ; see Table 4).

#### Environmental QoL

The environmental QoL domain showed significant variations related to living with the patient (*P* = 0.04), presence of diabetes mellitus (*P* = 0.01), education level (*P* = 0.002), employment status (*P* = 0.02) and monthly income ( 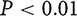 ), highlighting the impact of these factors on caregivers’ perception of their environmental QoL (Table 3). Moreover, the distance to the dialysis centre exhibited a weak negative correlation with environmental QoL (*r* = −0.128, *P* = 0.02), indicating that caregivers living farther from the centre tend to experience lower environmental QoL. Physical health showed a moderate negative correlation with environmental QoL (*r* = −0.298, 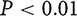 ). In addition, stress, anxiety and depression were all negatively correlated with environmental QoL (*r* = −0.325, 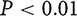 ; *r* = −0.344, 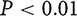 ; *r* = −0.369, 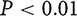 ), suggesting that higher levels of these psychological factors were associated with worse environmental QoL. Interestingly, both general QoL measures – QoL-Q1 (How would you rate your quality of life?) and QoL-Q2 (How satisfied are you with your health?) – showed strong positive correlation with environmental QoL.

## Discussion

The current study aimed to assess the levels of stress, anxiety and depression among family caregivers of Omani patients undergoing haemodialysis and to explore their association with QoL. The findings indicate that a significant percentage of caregivers reported experiencing various levels of stress, anxiety and depression, ranging from mild to extremely severe. This result is consistent with a descriptive study conducted in Brazil, which found a high prevalence of stress, anxiety and depression among family caregivers of patients undergoing haemodialysis.^7^ Various studies examining caregivers of patients with CKD undergoing haemodialysis in Greece, Saudi Arabia and Jordan have consistently shown a relationship between emotional difficulties – such as stress, depression and anxiety – and the caregiving role.^1,6,23^

The incidence of stress, anxiety and depression among caregivers can be influenced by various factors, including the caregiver’s relationship with the patient, the patient’s declining behaviour and mental state, gender and exposure to unpleasant life events.^1^ Furthermore, the demanding nature of caregiving, disruptions to sleep, health, social life and holiday preparations, as well as a sense of dependence on the dialysis facility, can contribute to the development of stress, anxiety and depression among caregivers.^1^ Other factors that contribute to psychological distress include low energy levels, burden levels, feelings of fatigue, financial difficulties, increased life obligations, limitations in social engagement and difficulty in coping with stress.^24,25^ In the current study this may be related to characteristics of the DASS. This tool is designed to be highly sensitive to differences in psychological distress and to capture a wide range of emotional symptoms. Because of its precision, it is particularly effective in identifying distress in populations facing ongoing challenges, such as caregivers. The combination of this sensitivity and the inherently stressful nature of caregiving likely explains why a significant number of participants scored in the ‘extremely severe’ range for depression and anxiety.

Findings in Oman also revealed that family members often volunteer to care for patients with chronic illnesses at home. This imposes additional responsibilities on caregivers, necessitating the acquisition of new skills and the ability to cope with the demanding nature of caregiving to fulfil their duties efficiently.^8,24^ However, these added responsibilities and challenges negatively affect the QoL of caregivers, who may also experience a higher mortality rate compared to those who do not provide care.^26^

In the current study, a significant negative association was observed between depression, anxiety and stress, on the one hand, and the QoL domains, on the other hand. Similar findings were reported by Pereira et al,^7^ Pio^27^ and Vovlianou et al.^28^ In general, taking on additional responsibilities (i.e. requiring more energy, time, attention and money) seriously increases caregivers’ levels of stress, anxiety and depression, adversely affecting their QoL, and vice versa. In other words, greater demand deteriorates the caregivers’ QoL, which, in return, increases their levels of stress, anxiety and depression. For example, the study findings highlighted that environmental QoL is relatively low, with significant negative correlations with stress, anxiety and depression. This QoL domain includes items related to safety, home environment and financial stability, highlighting the limited resources caregivers have to provide quality care to their patients within the home environment.

The relationship between environmental QoL and psychological symptoms is complex and influenced by various intervening factors. Recent studies have emphasised that improving financial security, access to healthcare and social resources can alleviate caregiver burden and enhance overall well-being. These findings signify the importance of targeted interventions to address both the environmental and psychological challenges faced by caregivers. Practical measures, such as financial assistance, improvements to home environments and the establishment of accessible support networks, can have a meaningful impact.

Furthermore, the research study found a statistically significant positive correlation between satisfaction with one’s own health and the mean scores of the physical, psychological and environmental domains of QoL.^29^ These findings align with the observations in the present study, highlighting the importance of caregivers’ subjective perceptions of their health in shaping their overall QoL.

Previous studies have consistently demonstrated negative associations between caregiver variables and QoL across multiple domains. Factors such as caregiver burden, physical health, stress, anxiety and depression were significantly associated with lower QoL in various domains, including physical, psychological, social and environmental.^8,30–32^ These findings underline the detrimental impact of caregiver variables on different aspects of caregivers’ QoL.

However, the current study’s findings regarding the relationship between the duration of caregiving and QoL contrast with those of Abdullah et al,^33^ who found an inverse relationship among caregiving strain, duration of caregiving and caregiver’s QoL. In their study, caregivers who had been providing care for more than 2 years experienced a 20–30% decline in QoL scores compared to those caregiving for less than 6 months, with the physical and psychological domains being particularly affected. This contrast may be explained by cultural and contextual differences between the two studies. In Oman, caregiving is often seen as a familial obligation, supported by extended family networks and community structures, which may help mitigate some of the stress typically associated with prolonged caregiving. In comparison, Abdullah et al’s study^33^ was conducted in a different cultural and healthcare setting, where caregivers may have less informal support and rely more on formal healthcare services. Moreover, the findings of the current study suggest that other factors, such as financial constraints, environmental challenges and a lack of formal support systems, may play a more significant role in shaping caregivers’ QoL than caregiving duration. Future research, particularly longitudinal studies across diverse cultural contexts, could provide valuable insights into these relationships and help identify effective strategies to support caregivers.

Consistent with the current study, research by Farzi^13^ found a significant relationship between the hours of care provided to patients by caregivers and the caregivers’ QoL. Another significant finding consistent with previous literature is that caregivers with their own health conditions – such as hypertension, depression and psychiatric morbidity – reported lower QoL scores. These findings align with previous studies, indicating that comorbid conditions are associated with variations in QoL domains.^4,26^

Moreover, studies have demonstrated the significant impact of income and financial strain on caregiver QoL, with lower-income caregivers experiencing poorer overall QoL across physical, psychological and social domains.^34–36^ These findings support the results of the current study, highlighting the importance of socioeconomic factors in shaping caregiver QoL.

### Recommendation

The results of the current study demonstrate the significant impact of psychosocial distress on every aspect of the QoL of caregivers providing care to patients undergoing haemodialysis. In the current study, it appeared that healthcare providers did not pay adequate attention to the psychological and physical needs of caregivers. Therefore, healthcare providers should periodically screen caregivers to ensure their overall well-being. While the high levels of stress, anxiety and depression observed in this study highlight the need for screening and intervention, it is essential to specify how such efforts might be implemented. Dialysis units, being closely connected to caregivers and familiar with their challenges, are well positioned to take the lead in these initiatives. Potential interventions could include creating structured support groups, offering educational programmes tailored to caregivers and collaborating with mental health professionals to provide counselling and emotional support. Future research should explore the feasibility of these strategies and evaluate their effectiveness to ensure that any screening efforts result in tangible benefits for caregivers.

In addition to addressing physical health, supportive interventions for caregivers should adopt a comprehensive approach to improve their economic, psychosocial and environmental needs. Comprehensive programmes considering these factors could have a major positive impact on their overall QoL, benefiting both patients and caregivers.

Future research should focus on cross-sectional comparisons of caregivers supporting patients on dialysis versus those with functioning transplants, as well as caregivers of individuals in comparable medical groups, such as stroke patients. Longitudinal studies observing changes in caregiver well-being before and after transplants would also provide valuable insights.

Furthermore, there is a case for testing straightforward interventions, such as the establishment of mutual support groups for caregivers. These groups could be conducted either in person or online, providing caregivers with a platform to share experiences, strategies and emotional support. Exploring the feasibility and impact of such approaches would be a meaningful direction for future research.

Finally, there is a need for qualitative studies to explore caregivers’ everyday living experiences and coping mechanisms. By addressing these research gaps, healthcare systems can better understand and meet the needs of this population, ensuring targeted, evidence-based support.

### Limitations

The main limitation of the current study is its design and reliance on self-reported data. A cross-sectional design prevents the establishment of causal relationships between the QoL domains and psychological distress variables. Longitudinal research is needed to ascertain the causality and directionality of these interactions. Furthermore, the use of self-reported data introduces the potential for recall bias.

In conclusion, this study highlights the significant psychological challenges faced by caregivers of patients undergoing haemodialysis, with many reporting high levels of stress, anxiety and depression. It also sheds light on the association between these psychological burdens and caregivers’ QoL, revealing how emotional distress can negatively affect various aspects of their well-being. Future research should focus on evaluating the feasibility of systematic screening for caregiver distress and understanding how best to develop accessible, evidence-based strategies to support them. By taking this approach, screening initiatives can be purposeful and lead to meaningful improvements in caregivers’ mental health and overall QoL.

## Data Availability

The data that support the findings of this study are available on request from the corresponding author, O.A.O. The data are not publicly available because they contain information that could compromise the privacy of research participants.
